# Fast-Evolving Mitochondrial DNA in Ceriantharia: A Reflection of Hexacorallia Paraphyly?

**DOI:** 10.1371/journal.pone.0086612

**Published:** 2014-01-27

**Authors:** Sérgio N. Stampar, Maximiliano M. Maronna, Marcelo V. Kitahara, James D. Reimer, André C. Morandini

**Affiliations:** 1 Universidade Estadual Paulista “Júlio de Mesquita Filho”, Laboratório de Biologia Aquática - LABIA, Faculdade de Ciências e Letras de Assis, Departamento de Ciências Biológicas, Assis, São Paulo, Brazil; 2 Universidade de São Paulo, Instituto de Biociências, Departamento de Zoologia, São Paulo, São Paulo, Brazil; 3 Universidade de São Paulo, Instituto de Biociências, Departamento de Genética e Biologia Evolutiva, São Paulo, São Paulo, Brazil; 4 Universidade de São Paulo, Centro de Biologia Marinha, São Sebastião, São Paulo, Brazil; 5 Molecular Invertebrate Systematics and Ecology Laboratory, Faculty of Science, University of the Ryukyus, Nishihara, Okinawa, Japan; Laboratoire de Biologie du Développement de Villefranche-sur-Mer, France

## Abstract

The low evolutionary rate of mitochondrial genes in Anthozoa has challenged their utility for phylogenetic and systematic purposes, especially for DNA barcoding. However, the evolutionary rate of Ceriantharia, one of the most enigmatic “orders” within Anthozoa, has never been specifically examined. In this study, the divergence of mitochondrial DNA of Ceriantharia was compared to members of other Anthozoa and Medusozoa groups. In addition, nuclear markers were used to check the relative phylogenetic position of Ceriantharia in relation to other Cnidaria members. The results demonstrated a pattern of divergence of mitochondrial DNA completely different from those estimated for other anthozoans, and phylogenetic analyses indicate that Ceriantharia is not included within hexacorallians in most performed analyses. Thus, we propose that the Ceriantharia should be addressed as a separate clade.

## Introduction

“DNA barcoding” is the usage of a standardized DNA region not only as a tool for fast and reliable identification of known species, but also to assist the detection of undescribed species [1]. Researchers utilize a short DNA sequence (∼700 bp) from the mitochondrial protein-coding gene cytochrome c oxidase subunit I (COI) that differs by several percent between even closely metazoan related species as an adequate “barcode” to distinguish species. Since then, the COI region has been widely used for DNA barcoding in many Metazoa, including cnidarians (see more in [2]). However, within cnidarians, the effectiveness of this marker as a species-level barcode is purported to be limited to Medusozoa [3] as it has levels of divergence (Kimura 2-Parameter, K2-P) within congeners greater than 20% in Medusozoa [2], while this divergence hardly exceeds 5% within anthozoan congeners [3]–[4]. Furthermore, other mitochondrial DNA markers broadly used – such as mitochondrial 16S rDNA – also have divergence values among anthozoan congeners significantly lower than those verified for medusozoans [3]. As such, it appears that the anthozoan mitochondrial molecular clock, most probably as a result of a mismatch repair system - e.g. a *MutS*-like protein encoded by the mt genomes of a number of octocorals [5]–[9], is slower than in most Metazoa. This low evolutionary rate has been inferred as an ancestral condition [3]; [10]–[11], although there is no evidence of this mismatch repair system in Hexacorallia mitochondrial genomes, or in other eukaryotes [12].

Within the “orders” that traditionally compose the Hexacorallia, only Ceriantharia does not have a representative with a complete mitochondrial genome determined to date. Phylogenetic studies of cerianthids based on mitochondrial and nuclear markers have resulted in reconstructions divergent from those based on morphology [11]; [13]–[15]. Thus, Ceriantharia is an “*incertae sedis*” group [13] within Anthozoa. In general, the phylogenetic reconstruction of higher taxonomic levels (i.e. class, order) demands molecular markers with “low” mutation rates (e.g. less than 1% divergence/million years) [16]. Therefore, the long (28S) and short (18S) nuclear ribosomal genes have been purported to be adequate [17] if they are conserved enough to produce unambiguous alignments and to provide an appropriate phylogenetic signal to define basal relationships [18]. In this study, nearly complete sequences from 18 and 28S and partial 16S ribosomal genes were used to reconstruct the cnidarian evolutionary history, and specifically focus on the relative position of cerianthids within the phylum. In addition, the genetic divergences of mitochondrial markers (i.e. partial 16S and COI sequences) were defined, and compared between Ceriantharia and other cnidarian groups, indicating a “fast-evolving mtDNA profile” (based on medusozoan data) in the former. In general, this study adds to the development of a more cohesive evolutionary scenario for Ceriantharia, defines a phylogenetic framework for Ceriantharia systematics, and proposes a new evolutionary scenario for the mutation history of mtDNA in the non-bilaterian Metazoa.

## Materials and Methods

### DNA Extraction, Polymerase Chain Reaction and Sequencing

Total DNA was extracted from individual cerianthid specimen tentacles (Table 1) using InstaGene® (Bio-Rad #732-6030) or the DNAdvance® kit (Agencourt® #A48705). PCR reactions and conditions followed under predefined conditions ([13]; primers used in the present study are listed in Table 2). Amplicons were purified using AMPure® kit (Agencourt® #A63881) following manufacturer’s instructions, and made ready for sequencing using the BigDye® Terminator v3.1 kit (Applied Biosystems #4337455; same primers and Tm temperature conditions as in PCR reactions). Sequencing was carried out on an ABI PRISM®3100 genetic analyzer (Hitachi), and resulting sequences were assembled and edited using Geneious™ 5.4.4 (Table 1). Ceriantharia sequences from mitochondrial (COI, 16S) and nuclear (18S, 28S) molecular markers were obtained from 12 of the 50 taxonomically recognized species [28].

**Table 1:** List of Ceriantharia species included in the present study.

Family	Organism	Obtained sequences	GENBANK-DDBJ/BOLD Number
**Arachnactidae**	***Isarachnanthus bandanensis***	16S/COI	**16S -** (JX125699) **COI** - (CMBIA097-11(BOLD))
	***Isarachnanthus maderensis***	16S/COI/18S	**16S** - (JX125670–72/79–80/82/85–87)/**COI** - (JX128313–14/22–23/25/28–33)/**18S** – AB859825
	***Isarachnanthus nocturnus***	16S/COI/28S/18S	**16S** - (JX125669/73–78/81/83–84/88–89/91–98)/**COI** - (JF915196–97/JX128315–21/24/26/27/34–42)/**18S** - AB859826/**28S** - AB859832
**Cerianthidae**	***Ceriantheomorphe brasiliensis***	16S/COI/18S/28S	**16S -** JF915193/**COI** - JF915195/**18S** – AB859823/**28S** – AB859831
	***Ceriantheopsis americanus***	16S/COI	**16S –** AB859834/**COI** – AB859839
	***Cerianthus membranaceus***	16S/COI/18S	**16S –** AB859837/**COI** – AB859843/**18S** – AB859824
	***Cerianthus lloydii***	16S/COI	**16S –** AB859838/**COI** AB859844
	***Pachycerianthus*** ** sp.1**	16S/COI/18S/28S	**16S –** AB859835/**COI** – AB859840/**18S** – AB859829/**28S** – AB859833
	***Pachycerianthus borealis***	16S	**16S** - U40288
	***Pachycerianthus magnus***	16S/COI/18S	**16S –** AB859836/**COI** – AB859841/**18S** – AB859828
	***Pachycerianthus fimbriatus***	COI/18S	**COI** – AB859842/**18S** –AB859827

**Table 2:** List of primers used to amplify/sequence Ceriantharia representatives in this study.

Marker	Primers	*Tm*	Reference
**COI**	LCO 1490+ HCO2198	49°	[19]
	LCO 1490+ HCOout	48°	[20]
	LCO 1490+ HCOCato	48°	[21]
			
**16S**	CB1+ CB2	56°	[22]
	CB1+ R [BR]	54°	[23]
	F1Mod+CB2	56°	[22]
			
**18S**	18C+18Y	55°	[24]
	18A+18L	55°	[24]
	18O+18B	55°	[24]
			
**28S**	5S-R635, F635–R1630, F1379–R2077,F2076–R2800, F2800–R3264	63°	[25] [26] [27]

### Mitochondrial Evolutionary Divergence between Ceriantharia Species and Genera

**Figure 1:**
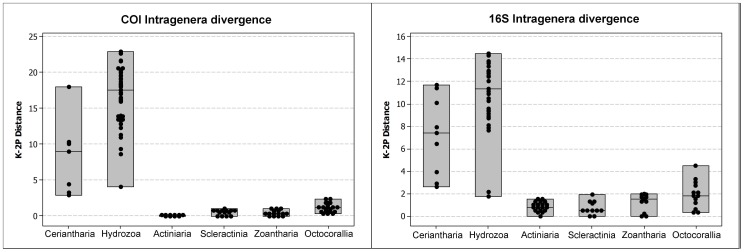
Estimates of evolutionary divergence in major Anthozoan lineages from mitochondrial molecular markers (intrageneric level, Hydrozoa considered as an outgroup for discussion). Right graph: estimates of evolutionary divergence (Kimura 2-parameter model) of COI among congeners of Ceriantharia (n = 9), Hydrozoa (n = 10), Actiniaria (n = 5), Scleractinia (n = 7), Zoantharia (n = 7) and Octocorallia (n = 6), (n = number of species examined). Left graph: estimates of evolutionary divergence (Kimura 2-parameter model) of 16S ribosomal DNA among congeners of Ceriantharia (n = 9), Hydrozoa (n = 11), Actiniaria (n = 9), Scleractinia (n = 7), Zoantharia (n = 6) and Octocorallia (n = 6), (n = number of species examined).

**Figure 2:**
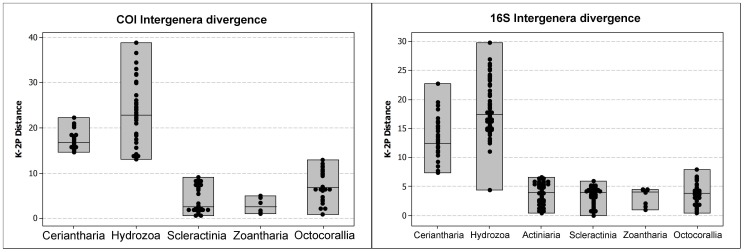
Estimates of evolutionary divergence in major Anthozoan lineages (intergenera level, Hydrozoa considered as outgroup for discussion) from mitochondrial molecular markers. Right graph: estimates of evolutionary divergence (K2-P) of COI between species of different genera in Ceriantharia (n = 8), Hydrozoa (n = 12), Scleractinia (n = 10), Zoantharia (n = 4) and Octocorallia (n = 8) (n = number of species examined). Left graph: estimates of evolutionary divergence (Kimura 2-parameter model) of 16S ribosomal DNA between species of different genera in Ceriantharia (n = 9), Hydrozoa (n = 13), Actiniaria (n = 12), Scleractinia (n = 11), Zoantharia (n = 5) and Octocorallia (n = 11) (n = number of species).

Evolutionary distances of mitochondrial (16S rDNA and COI) genes from Ceriantharia were analyzed within congeners (*Isarachnanthus* – see Table 3) and between genera in MEGA5 software [29], using Kimura’s two-parameter model of base substitution (K2-P) in order to calculate their respective genetic distances. In addition, K2-P genetic distances were also estimated for other anthozoan clades, and hydrozoans were considered as “outgroup” for discussion. The distances obtained for both mitochondrial markers are compiled in four graphs in two figures, of which one are from congeners (Figure 1), and another from species of different genera (Figure 2). The complete datasets, including sequences obtained from GenBank, are compiled in the supplementary material (Table S1).

**Table 3:** Estimates of average evolutionary divergence over sequence pairs within *Isarachnantus* representatives.

Molecular Marker	Species	Sample Size	Within Species		Between Species	
			*d* (%)	S.E.	*d* (%)	S.E.
**16S**	*I. maderensis*	9	0.5	0.002	6.0	0.01
	*I. nocturnus*	20	0.0	0.000		
**COI**	*I. maderensis*	11	0.0	0.000	8.9	0.01
	*I. nocturnus*	21	0.1	0.000		

The number of base substitutions per site from averaging over all sequence pairs within each group is shown. *d* – Distance in % and S.E. – Standard error estimates were obtained by a bootstrap procedure (500 replicates).

### Phylogenetic Inferences and Hypotheses Testing

Representing all accepted cnidarian clades except Myxozoa (*sensu* [11]; Text S1), 18S and 28S rDNAs sequences were used to reconstruct the cnidarian evolutionary history. In order to avoid the effects of an excess of missing entries on a combined analysis, each molecular marker was analyzed with a single gene analysis approach. In order to avoid any limitation on restricted results from a certain method of alignment and phylogenetic method, we analyzed our 18S and 28S datasets with several alignment methods, masking data and all standard phylogenetic reconstruction methods: (1) “static” defined homology applying consistency scores (MAFFT, [30]) and estimated secondary structure (RNAsalsa; [31]); (2) “dynamic” defined homology applying probabilistic approaches with indel model (Bali-Phy; [32]), gaps treated as missing data (MAFFT; [30] and OPAL; [33] in Saté; [34]), and through several transformation costs in parsimony (POY; [35]). To consider the possible influence of gaps, and because some positions with noise signal in molecular datasets may result in topologies with phylogenetic artifacts, we made extra datasets for analyses: (1) gaps coded as an additional binary datamatrix (for probabilistic based methods; Fastgap; [36]) and (2) highly variable positions were detected as potentially noisy positions, and were masked (filtered) using Aliscore [37].

**Figure 3:**
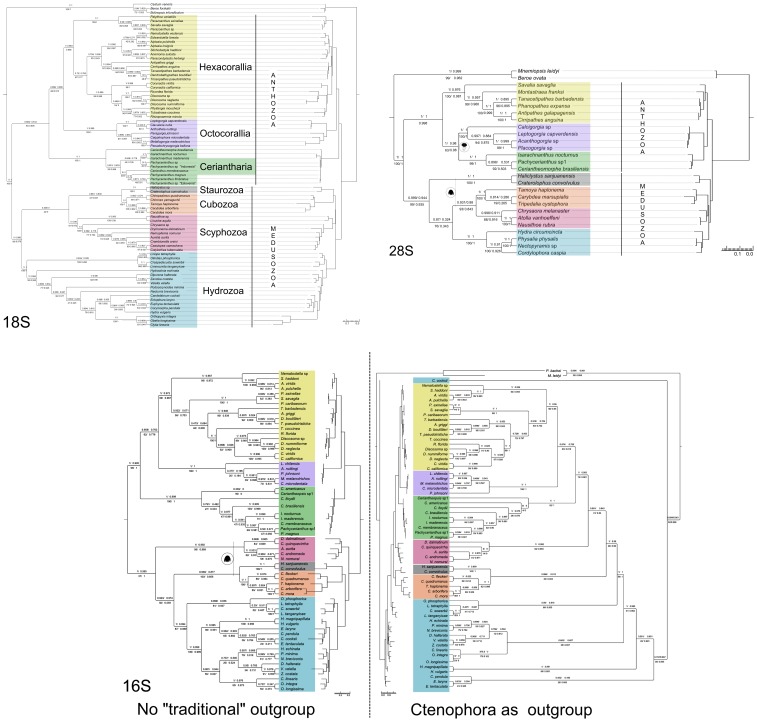
ML phylogenies of Cnidaria based on ribosomal molecular markers 18S (upper left), 28S (upper right) and 16S datasets (lower center). Major cnidarian lineages are in cladograms and phylograms for Medusuzoa clades (Hydrozoa, Scyphozoa, Cubozoa, Staurozoa) and Anthozoa (Hexacorallia, Hexacorallia, with special emphasis with Ceriantharia (green). Divergent results in the 18S phylogeny (considering the major clades) are shown in 16S (no Ctenophora sequences in analysis) and 28S trees (jellyfish icon for Medusozoa clades, polyp icon for Anthozoa clades). Supports values are aBAYES and aLRT (parametric, upper branch) together with BS and SH-like (non parametric, lower branch) in clockwise fashion. Each dataset was aligned in MAFFT and phylogeny estimated in RAxML; support values were calculated in RAxML (BS) and PhyML (aBAYES, aLRT and SH-like; see Table S2 for details in datamatrices, software and parameters).

Considering the phylogenetic reconstructions methods, we analyzed the datasets under Maximum Likelihood (RAxML, Saté; [38]; [34]), Bayesian inference [MrBayes, Bali-Phy; [39], [32]) and Maximum Parsimony (TNT, POY; [40], [35]; see Text S1 and for Table S3 for full parameterśs information on all thirteen phylogenetic reconstructions’ approaches.) To evaluate nodal support and to detect if support values were biased, two parametric (aLRT and aBAYES) and two non-parametric (Bootstrap, BS and SH-aLRT) techniques were applied in MAFFT+RAxML results [38]; [41]). Bootstrap values were computed on RAxML v7.3.2 (500 pseudoreplicates, same parameters as the original phylogenetic analysis) and additional statistical tests were performed using PhyML v3.0.1 [42]; [43]) under the same parameters as the original MAFFT+RAxML inferences (Figure 3). As additional datasets, representing mitochondrial DNA we analyzed partial sequences from the mitochondrial ribosomal gene 16S for 63 cnidarian species with outgroups as used in previous works (see Text S1, Table S1 and Table S2) for final rooting, and a dataset including two extra Ctenophora species for final outgroup consideration (both analyses with no masking option). Sequences were aligned in MAFFT (parameter: auto), phylogeny estimated in RAxML (GTR DNA model, 125 replicates) and support values estimated as in 18S and 28S studies. A combined dataset was created for 18S and 16S sequences, with basic similar results to single gene 16S analysis (see Table S1 and Figure 3 and Figure S1 for sampled species and phylogenetic results). Different data treatment and phylogenetic analysis approaches were additionally tested on a COI dataset, but no major recognizable cnidarian clades were recovered (these results were not included in our main results: see Figure S2 and Figure S3 for results and analyses’ details).

**Figure 4:**
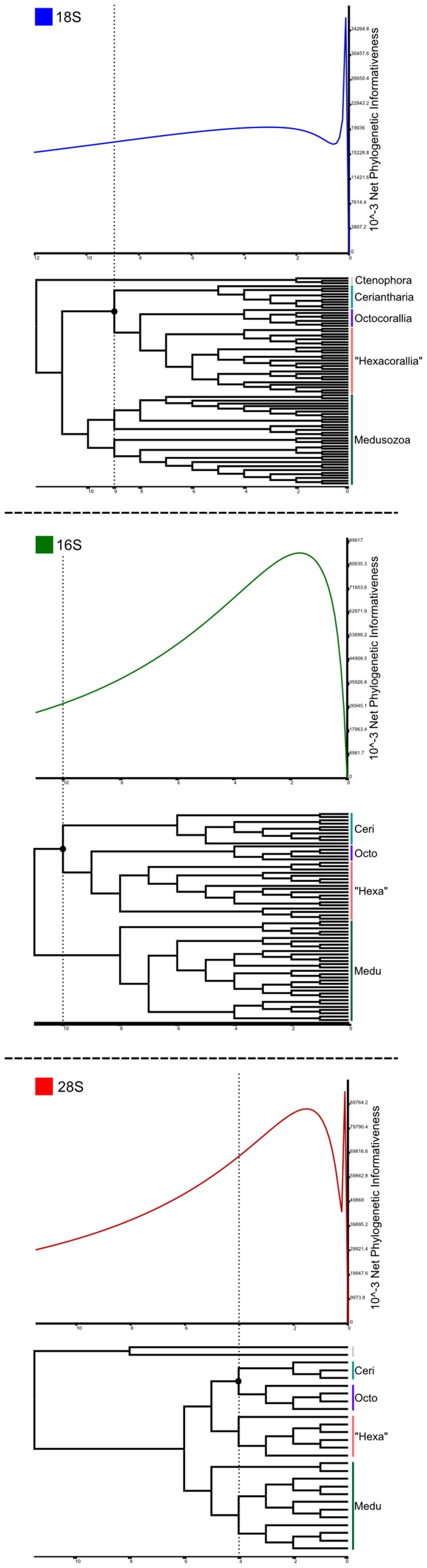
Phydesign curves - Phylogenetic Informativeness profiles from the 18S, 16S (no Ctenophora sequences in analysis), and 28S datasets, computed with their original datasets and results from the MAFFT+RAxML results. The x-axis represents topologies considering their nodes as epoch units, the y-axis represents the Net Phylogenetic Informativeness value for each molecular marker along the topologies.

To analyze the phylogenetic signal profile along the recovered phylogenies in figure 3, the net approach test was performed using PhyDesign (Figure 4; [44]). Finally, to test alternative systematic proposals and our own main results, considering the phylogenetic position of Ceriantharia, we computed AU and other phylogenetic hypothesis tests using consel ([45]; same input datasets and results from the MAFFT+RAxML analysis; Table 4).

**Table 4:** Phylogenetic results considering all the analyzed ribosomal datasets and methods, showing the recovery of major clades in Cnidarian systematics proposals in our topologies and literature.

Tree Topology (phylogenetic hypotheses)	Static homology strategy							Dynamic homology strategy					
	ML (x4)			BY (x2)		MP (x30)		ML (x2)		BY		MP(x18)	
	18S	28S	16S	18S	28S	18S	28S	18S	28S	18S	28S	18S	28S
Anthozoa monophyletic	(1,2,3)	YES	YES	YES	YES	2/30	YES	(1)	YES	no	YES	6/18	YES
**Ceriantharia sister group of rest of** **Hexacorallia**	no	no	no	no_poli	no	no	2/30	no	no	no	no	no	4/18
**Ceriantharia sister group of Octocorallia**	no	YES	no	no_poli	YES	1/30	19/30	no	YES	no	YES	no	9/18
**Ceriantharia sister group of rest of Cnidaria**	ali(4)	no	no	no_poli	no	27/30	no	ali(2)	no	YES	no	7/18	no
**Ceriantharia sister group of rest of Anthozoa**	(1,2,3)	no	YES	no_poli	no	no	6/30	(1)	no	no	no	2/18	4/18
Octocorallia monophyletic	YES	YES	YES	YES	YES	29/30	YES	YES	YES	YES	YES	11/18	YES
Rest of the “Hexacorallia” clade monophyletic	YES	YES	YES	YES	YES	YES	YES	YES	YES	YES	YES	14/18	15/18
Octocorallia sister group of Medusozoa	ali(4)	no	no	no	no	4/30	no	ali(2)	no	no	no	4/18	no
Medusozoa monophyletic	YES	YES	YES	YES	YES	28/30	26/30	YES	YES	YES	YES	11/18	YES
Staurozoa, Scyphozoa, Cubozoa and Hydrozoa monophyletics	YES	YES	YES	YES	YES	23/30	YES	YES	YES	YES	YES	11/18	16/18
Staurozoa sister group of rest of Medusozoa	no	YES	no	no	YES	1/30	24/30	ali(2)	ali(2)	no	YES	no	6/18
Staurozoa sister group of Scyphozoa	(3) & ali(4)	no	no	no	no	4/30	no	no	no	no	no	no	no
Staurozoa sister group of Cubozoa	(1,2)	no	YES	YES	no	9/30	no	(1)	no	YES	no	13/18	no

Major results considering Ceriantharia’s position are shown in blue or black font. Results are presented from the static and dynamic homology strategies, considering the 18S, 16S (no Ctenophora species in analysis) and 28S datasets. Static homology strategy, ML (x4) = Maximum Likelihood results considering 4 different analytical strategies: (1) MAFFT+RAxML, (2) RNAsalsa+RAxML (with and without secondary structure info included in these analysis), (3) gap coded as binary data matrix (RNAsalsa+FastGap+RAxML), (4): (ali: RNAsalsa+aliscore+RAxML); Static homology strategy, BY (x2) = Bayes results considering 2 different analytical strategies: (1) no gap coded (RNAsalsa+MrBayes), (2) gap coded (RNAsalsa+FastGap+MrBayes); Static homology strategy, MP = maximum parsimony results considering 30 different analytical strategies, being divided as 15 analyses with different cost weight regimes (3 of them treating gaps as missing data: RNAsalsa+TNT) and other 15 similar cost weight regimes, after alignment masking with (RNAsalsa+aliscore+TNT); Dynamic Homology strategy, ML (x2) = Maximum Likelihood results considering 2 different analytical strategies: (1) SATé (CLUSTALW+RAxML) and (2) a 18S filtered datamatrix (ali: RNAsalsa+aliscore+(SATé = MAFFT+RAxML)); Dynamic Homology strategy, BY = Bayes results from a dynamic homology analysis (BAli-Phy); no_poli = basal politomy in Anthozoa related to major recovered clades (Ceriantharia, Octocorallia and “Hexacorallia). In cases of all result/s being the considered hypotheses: YES (black box); most of results of the considered hypotheses were equal or >50% of total analyses: number of positive (dark grey); most of results considered the hypotheses were <50% of total analyses: number of positive results (light grey).

## Results

### Mitochondrial Genetic Diversity

While the genetic distance estimates for COI and 16S within Ceriantharia congeners ranged from 3 to 17% and 3 to 12%, respectively (Figures 1 and 2), values for all other anthozoan groups excepting Octocorallia were estimated to be less than 1% (COI) to 2% (16S). Octocorallian congeners slightly exceeded these values, with approximately 2% and 4% divergences within COI and 16S sequences, respectively. In general, the divergence values estimated for ceriantharians showed a range most similar to those from hydrozoans (medusozoan example) (intrageneric divergence: COI/16S 3–17%/3–12% (Ceriantharia) and 4–23%/2–14% (Hydrozoa)). Furthermore, the estimated COI genetic divergence between genera in every major anthozoan group (Octocorallia and Hexacorallia) was lower than the divergence observed for ceriantharian congeners. Most anthozoans had intergeneric divergences below 10% while the estimated divergence in ceriantharians was between 14 and 22% (Figure 3). However, the calculated 16S divergence between species of different genera showed less difference between Ceriantharia and other anthozoans, as observed values in Ceriantharia ranged from 7 to 23%, while other anthozoan groups ranged from 0 to 7%.

An example of the genetic divergence of related congeneric species is the divergence values of *Isarachnanthus* spp. in the Atlantic Ocean ( = 6–9%), which are higher than levels reported from similar comparisons in other anthozoans. The estimated divergence between several specimens of *I. nocturnus* and *I. maderensis* was based on multiple specimens and is considered reliable (Table 3).

### Phylogenetic Position of Ceriantharia

The final alignments consisted of 73 (18S rDNA), 63 (mitochondrial 16S rDNA) and 25 (28S rDNA) cnidarian species, with 1992, 1184 and 3013 positions, respectively (MAFFT results). Instead of Porifera, Ctenophora was chosen as outgroup in all studies, as fewer gaps were required in order to align their sequences together with cnidarian sequences.

**Figure 5:**
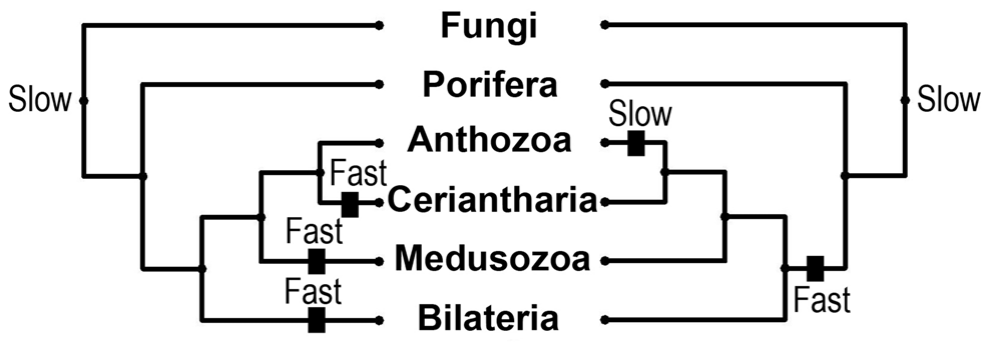
Graphic representation of possible evolutionary scenarios for slow mtDNA evolution in Anthozoa, modified from [39]. Scenario A: fast evolution originated in the Ceriantharia, Medusozoa and Bilateria independently (adapted from [43]). Scenario B: fast mtDNA evolved as a unique event (Cnidaria+Bilateria) but was subsequently lost in Anthozoa (not including Ceriantharia).

Results of the phylogenetic reconstructions are show in Figures 3 and 5 and summarized below.

Both 18S and 28S genes recovered monophyletic Anthozoa and Meduzozoa with a sister relationship in almost all results (Figures 3 and 4), although the Hexacorallia monophyly suffered due to Ceriantharia’s relative position. In most 18S reconstructions, all anthozoan and medusozoan lineages (i.e. Octocorallia, Hexacorallia, Scyphozoa, Staurozoa, Cubozoa, and Hydrozoa) were recovered as monophyletic groups. However, Ceriantharia was recovered as a sister group to Anthozoa, or in some cases, as a sister group of Cnidaria (Table 4). Ceriantharia, which has been taxonomically placed within Hexacorallia, was recovered as such only with dynamic homology with low CI and RI indexes if compared to other MP-POY topologies from the same dataset. A sister-like relationship between Ceriantharia and Octocorallia was also recovered in MP topologies where gaps were treated as missing data (TNT analysis), or in ML and MP with filtered datasets (RAxML and POY results; Table 4; except for these cases, Ceriantharia was recovered as a sister group to Cnidaria).

The monophyly of both Anthozoa and Medusozoa, and their respective major component groups, were also recovered using 28S rDNA (Figure 3, Table 4). However, in general, the main topology differed from that of 18S rDNA as Ceriantharia occupied a sister-like position to Octocorallia, with high statistical support in all but non-parametric bootstraps. MP topologies that recovered Ceriantharia as sister group of Hexacorallia (traditional placing) had several unexpected results – for example, non-monophyletic Scyphozoa and Staurozoa as sister groups of Hydrozoa. As in 18S rDNA, these MP topologies had lower CI and RI values compared to results using different methods (Table S2). The 16S dataset including two ctenophore sequences had a loss of monophyly of the Medusozoa group and some well-recognized cnidarian clades (e.g., Hydrozoa), with Ceriantharia grouped with the rest of the anthozoan lineages (but not as sister group of the rest of Hexacorallia), possibly representing a case of basal long branch attraction for portions of the sampled Hydrozoa and Ctenophora sequences. For the 16S dataset we rooted the 16S phylogeny considering Medusozoa and Anthozoa as monophyletic groups, and this analysis had highly similar results as to the 18S ML analysis and rooted with ctenophore sequences (Figure 3). It is important to note that the late definition of rooting does not affect analyses in ML studies nor phylogenetic relationships between the terminal trees. Due to better support values and more similar clade relationships for major cnidarian lineages with 18S and 28S results, we focused on the 16S dataset and their ML result that did not include ctenophoran sequences for Consel and PhyDesign analysis. Consel recovered low support (negative significance value - less than 0.05 of p-value) for Ceriantharia as sister group of Hexacorallia compared to alternative systematic scenarios in both 18S and 16S analyses (Table 5). Based on PhyDesign outcomes, it is believed that the 28S rDNA has more phylogenetic signal than the 18S rDNA, and consequently is more adequate for Cnidaria higher rank reconstruction (Figure 4). However, it is important to note that these results may be biased due to the different number of species included in each gene dataset (and consequently different number of epochs in each topology; Figure 4).

**Table 5:** Hypothesis testing results, considering Bayesian posterior probability calculated by the BIC approximation (PP) and results based on p-values; significance level result for AU (>0.05), rejecting the Hexacorallia clade in the traditional proposal remarked in blue).

Phylogenetic hypotheses (18S datamatrix)	AU	PP	KH	SH	WKH	WSH
**Ceriantharia sister group of rest of Anthozoa**	**0.861**	**0.901**	**0.812**	**0.846**	**0.812**	**0.844**
Ceriantharia sister group of Octocorallia	**0.214**	**0.054**	0.188	0.190	0.188	0.329
Hexacorallia “monophyletic”	**0.151**	**0.045**	0.169	0.173	0.169	0.306
**Phylogenetic hypotheses (16S datamatrix)**	**AU**	**PP**	**KH**	**SH**	**WKH**	**WSH**
**Ceriantharia sister group of rest of Anthozoa**	**0.760**	**0.953**	**0.746**	**0.825**	**0.746**	**0.799**
Ceriantharia sister group of Octocorallia	**0.273**	**0.041**	0.254	0.268	0.254	0.370
Hexacorallia “monophyletic”	**0.035**	**0.06**	0.101	0.114	0.101	0.192
**Phylogenetic hypotheses (28S datamatrix)**	**AU**	**PP**	**KH**	**SH**	**WKH**	**WSH**
**Ceriantharia sister group of Octocorallia**	**0.873**	**1.0**	**0.871**	**0.93**	**0.871**	**0.909**
Ceriantharia sister group of rest of Anthozoa	**0.137**	**0.0**	0.13	0.132	0.129	0.208
Hexacorallia “monophyletic”	**0.003**	**0.0**	0.021	0.022	0.021	0.045

The original phylogenetic results from these datasets and their results, as well main results from the approximately unbiased test (AU, non parametric test) and posterior probabilities values (PP, parametric test) are shown in bold. The 18S and 28S datasets were aligned with RNAsalsa and 16S dataset (no Ctenophora species in analysis) was aligned with MAFFT, with no data masking in all cases. Other tests: Kishino-Hasegawa test (KH); Shimodaira-Hasegawa test (SH); weighted Kishino-Hasegawa test (WKH); weighted Shimodaira-Hasegawa test (WSH).

Overall, our results from both nuclear and mitochondrial ribosomal gene analysis corroborate that Ceriantharia is a monophyletic and basal independent lineage, but indicates that it does not have a direct relationship to other hexacorallian orders. Thus, these results suggest that Ceriantharia should be ranked as a separate subclass within Anthozoa.

## Discussion

### Anthozoan Phylogenetics and Genetic Divergence among Cnidarian Species and Genera

According to [3], “the tempo of evolution in anthozoan mitochondrial genes appears to be at least 10–20 times lower than the standard mitochondrial clock based on vertebrate sequences, which averages a sequence divergence of 1–2%/Myr [46]–[49]…”. With the use of a number of statistical methods, [50] also found that the anthozoan intra- and interspecific COI variations were ≤6% or invariant (in 98% of the analyzed species). The genetic divergence of ceriantharian mitochondrial markers would therefore be expected to follow the slow rate of differentiation as detected in other groups currently classified as anthozoans [51]. However, an overview of our results suggests a much faster rate. For both analyzed mitochondrial markers (COI and 16S rDNA), the genetic divergence rates estimated between Ceriantharia congeners are more similar to those from Hydrozoa than to Anthozoa, suggesting that the mitochondrial clock in Ceriantharia has almost the same divergence rate as in Medusozoa and other Bilateria groups (see also [13]).

Therefore, the above statement of slow mitochondrial evolution in Anthozoa is accurate only if **Ceriantharia is not included within Anthozoa classification.** In fact, this hypothesis is not unusual. Based on nuclear molecular markers, [15] and [11] showed that Ceriantharia is not within Hexacorallia. Another study also stressed that Ceriantharia could be a hexacorallian outgroup [52], but its position was not further discussed. In addition, analyses of complete mitochondrial genomes indicate that the phylogenetic position of Ceriantharia is unstable and not well defined (e.g. sister group of Hexacorallia) [53]. For discussion on the historical context of molecular data and Ceriantharia’s evolutionary position over the last twenty years see (Text S1).

More recently, however, a new body of data is changing the scenario previously proposed for the origin of the rapid evolution of mtDNA [50]. In summary, there are two most parsimonious explanations for the slow mtDNA evolution in anthozoans (see Figure 2 in [50]). However, both scenarios must be modified to accommodate the Ceriantharia results presented herein. The first scenario (Figure 5, reconstruction A) hypothesizes independent origins for the rapid evolution of mtDNA in Medusozoa and Bilateria. However, it is necessary to include an additional step (origin of the rapid evolution of mitochondrial DNA) before Ceriantharia, and consequently this reconstruction is no longer most parsimonious. In the second scenario (Figure 5, reconstruction B) there is no necessity to add an extra step, making it the most parsimonious scenario; namely that anthozoans (not including Ceriantharia) decreased their mtDNA rate of evolution.

Corroborating the findings from [15], the phylogenetic “uniqueness” of Ceriantharia in relation to anthozoans has major implications not only in basal metazoan mtDNA evolution but also in cnidarian evolutionary history. The inclusion of Ceriantharia data in molecular clock phylogenetic reconstructions may influence estimates for the appearance of Cnidaria. It may also have effects on the stability of the position of Cnidaria in broader studies, where the monophyly of the group (and major lineages, like Anthozoa and Medusozoa) are compromised by potential incomplete taxon sampling [53]–[55]. From the current state of knowledge, it is possible to infer that Ceriantharia does not belong to Hexacorallia and thus represents a new subclass. However, the exact phylogenetic position of Ceriantharia is still debatable. Our study indicates that, based on a broader sampling of the group, Ceriantharia is most likely a sister group to Anthozoa. However, a sister-like relationship between Ceriantharia and Octocorallia cannot be discarded and further analyses are needed to clarify this matter.

### Futures Prospects on Cnidarian mtDNA Evolutionary Genetics

A better understanding of the mechanisms related to the shifts of mtDNA evolutionary rates is a challenging task in current cnidarian studies [56]–[58]. There are several possible historical influences on qualitative aspects of mtDNA on this group, such as genome linearization and fragmentation, horizontal gene transfer from a non-bilaterian species (e.g., HGT, *mtMutS* gene), and gene arrangement (e.g., [59]–[61]). Recombination phenomena have been invoked to explain gene rearrangements in Octocorallia, and plasmid insertion to explain mtDNA linearization in Medusozoa [60]. The connection between genome components and rearrangements with mutation rates is not a trivial matter [62]–[65], as it is proposed to have been involved as one of the main mechanisms on evolutionary genetics of linear genomes [66]. Nonetheless, the theory of a mtDNA mismatch repair gene in Octocorallia as the mechanism explaining slow rates of genetic divergence does not explain the slow rates exhibited in Hexacorallia (Figures 1–3). This consideration reinforces the “classic” relationship proposed between Octocorallia and Hexacorallia lineages, differing from phylogenetic analyses based on mitochondrial data linking Octocorallia as sister group of Medusozoa (Anthozoa not monophyletic: [53]; [67]. Thus, more extensive studies on mitochondrial genome arrangement and how it is related to mutation/evolutionary patterns are needed in Hexacorallia. Additional efforts should also be undertaken to increase ceriantharian taxon sampling, evaluation of different cnidarian outgroups (e.g., Porifera vs Ctenophora), together with a thorough evaluation of (1) data treatment to define homology, (2) reconstruction parameters and methods, (3) nodal support, (4) hypotheses testing and the (5) recovered phylogenetic signal (e.g. [68]–[69]; [34]).

Recently [70] suggested that almost 90% of the Ceriantharia species are already known. However the authors neglected to address the existence of cryptic species, species with disjunct distributions (e.g. *Ceriantheomorphe brasiliensis*, *Ceriantheopsis americanus*), and oceanic areas with no data (e.g. deep sea); additionally, the evolutionary position of Ceriantharia should be clarified because of their possible reflection of basal adaptive radiation in cnidarians, considering their asymmetric conditions on sister clades, based on (1) number of extinct derived species, (2) genetic divergence and (3) morphological-ecological traits, that altogether could represent signals of adaptative events [67] [71]. Finally, we emphasize that the inclusion of Ceriantharia in Hexacorallia should no longer be accepted and this group should be elevated to subclass.

Thus the class Anthozoa should be divided into three subclasses;

Hexacorallia,
Octocorallia
Ceriantharia subclass nov.

## Supporting Information

Figure S1 **ML cladogram of Cnidaria based on a combined dataset (18S+16S ribosomal molecular markers partitions).** Supports values are aBAYES and aLRT (parametric, upper branch) together with BS and SH-like (non parametric, lower branch) in clockwise direction. Each dataset was aligned in MAFFT and phylogeny estimated in RAxML (independent branch length calculated for every molecular marker partition); support values were calculated in RAxML (BS) and PhyML. Data treatment and basic parameters (e.g., number of replicates) were similar to individual ML analysis for 18S dataset (MAFF+RAxML analysis; see Table S2 for details in datamatrices, software and parameters).
(TIF)

Figure S2 **ML phylograms of evolutionary relationships presented in** **figure 3** **, considering ribosomal molecular markers analysis (A: nuclear 18S; B: mitochondrial 16S (no Ctenophora species in analysis); C: nuclear 28S).**
(TIF)

Figure S3 **ML phylograms from different analysis datasets for cytochrome oxidase I (COI) cnidarian sequences estimated in Garli (100 replicates;** [**72**] **).** Partition analysis with PartitionFinder [73] defined two basic partitions as optimal to estimate gene phylogeny for COI (all codon positions): first and second position (partition 1, model TVM+G) and third position (partition 2, model SYM+G); then both partitions (Figure A) and partition 1 only (Figure B) were analyzed. Trying to overcome molecular saturation (non-phylogenetic related heterogeneity), the COI dataset was filtered at two different intensity levels: treating gaps as missing data (“Aliscore -N” strategy; Figure C) and a more intense approach (“Aliscore -N -r -w4”; Figure D). The COI dataset (Genbank IDs presented in terminal’s names) was originally aligned in MAFFT (codon frame checked); the root position was defined *a posteriori* (random position; no effect on ML analysis).
(TIF)

Table S1 **List of DNA sequences species used in this study considering the 18S, 28S and 16S datasets (new sequences remarked in blue).**
(XLSX)

Table S2 **Software and analytic details (data treatment and parameters) for every analysis applied in this study for 18S and 28S datasets (see** **Table 4** **for major results compilation).**
(XLSX)

Table S3 **Results from TNT analysis (18S and 28S datasets), considering original and masked alignments (RNAsalsa+aliscore), in all analyzed cost weight transformations (indel:transitions:transversions).**
(XLSX)

Text S1 **Discussion on evolution and previous systematic considerations of Ceriantharia and Anthozoa.**
(DOCX)
