# Supplementary figures and images for: Fast-Evolving Mitochondrial DNA in Ceriantharia: A Reflection of Hexacorallia Paraphyly?
Source: PLoS One. 2014 Jan 27;9(1):e86612. doi: 10.1371/journal.pone.0086612 (PMC3903554; doi:10.1371/journal.pone.0086612)

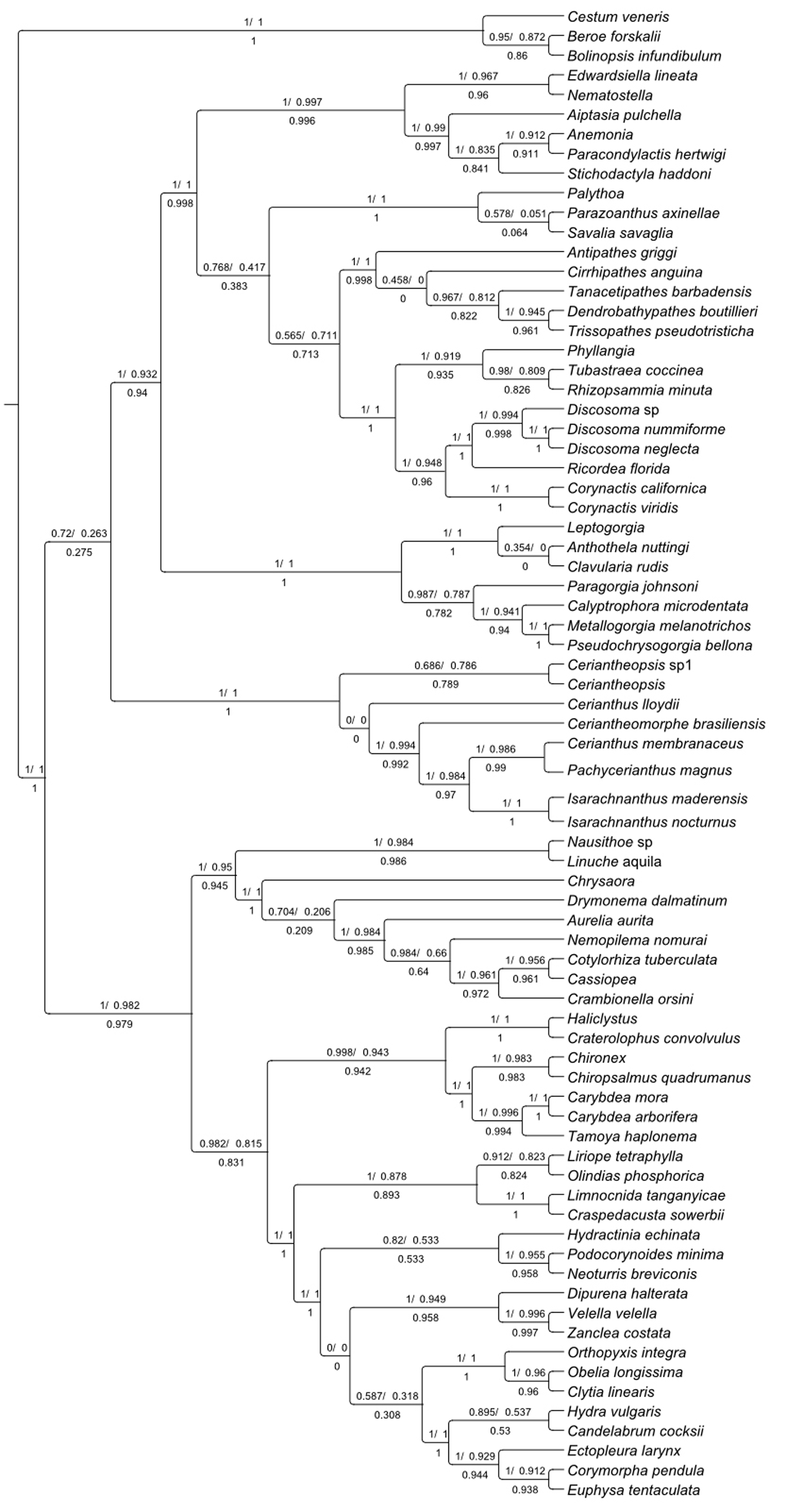

Supplement: Figure S1 — ML cladogram of Cnidaria based on a combined dataset (18S+16S ribosomal molecular markers partitions). Supports values are aBAYES and aLRT (parametric, upper branch) together with BS and SH-like (non parametric, lower branch) in clockwise direction. Each dataset was aligned in MAFFT and phylogeny estimated in RAxML (independent branch length calculated for every molecular marker partition); support values were calculated in RAxML (BS) and PhyML. Data treatment and basic parameters (e.g., number of replicates) were similar to individual ML analysis for 18S dataset (MAFF+RAxML analysis; see Table S2 for details in datamatrices, software and parameters). (TIF) [file pone.0086612.s001.tif]

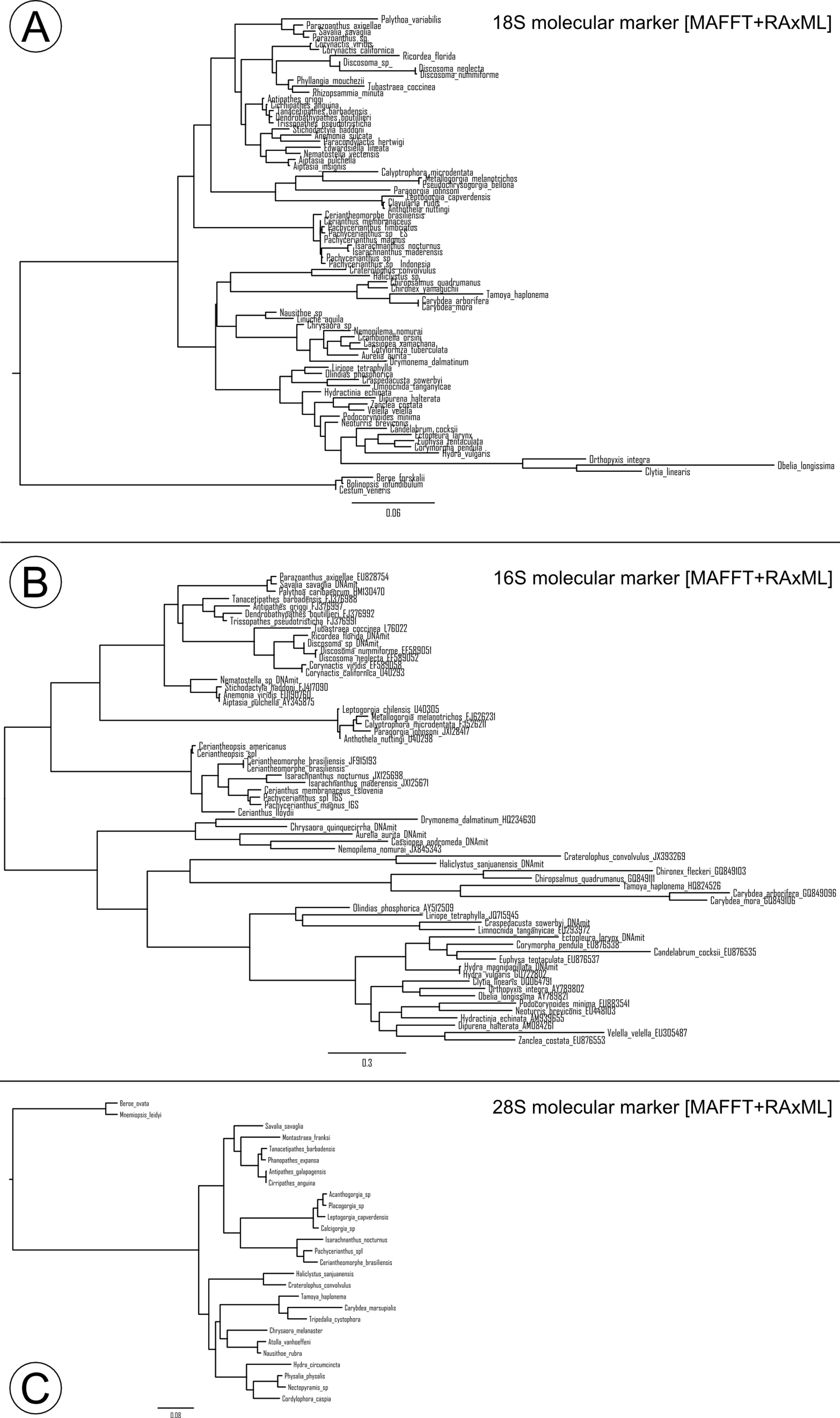

Supplement: Figure S2 — ML phylograms of evolutionary relationships presented in figure 3 , considering ribosomal molecular markers analysis (A: nuclear 18S; B: mitochondrial 16S (no Ctenophora species in analysis); C: nuclear 28S). (TIF) [file pone.0086612.s002.tif]

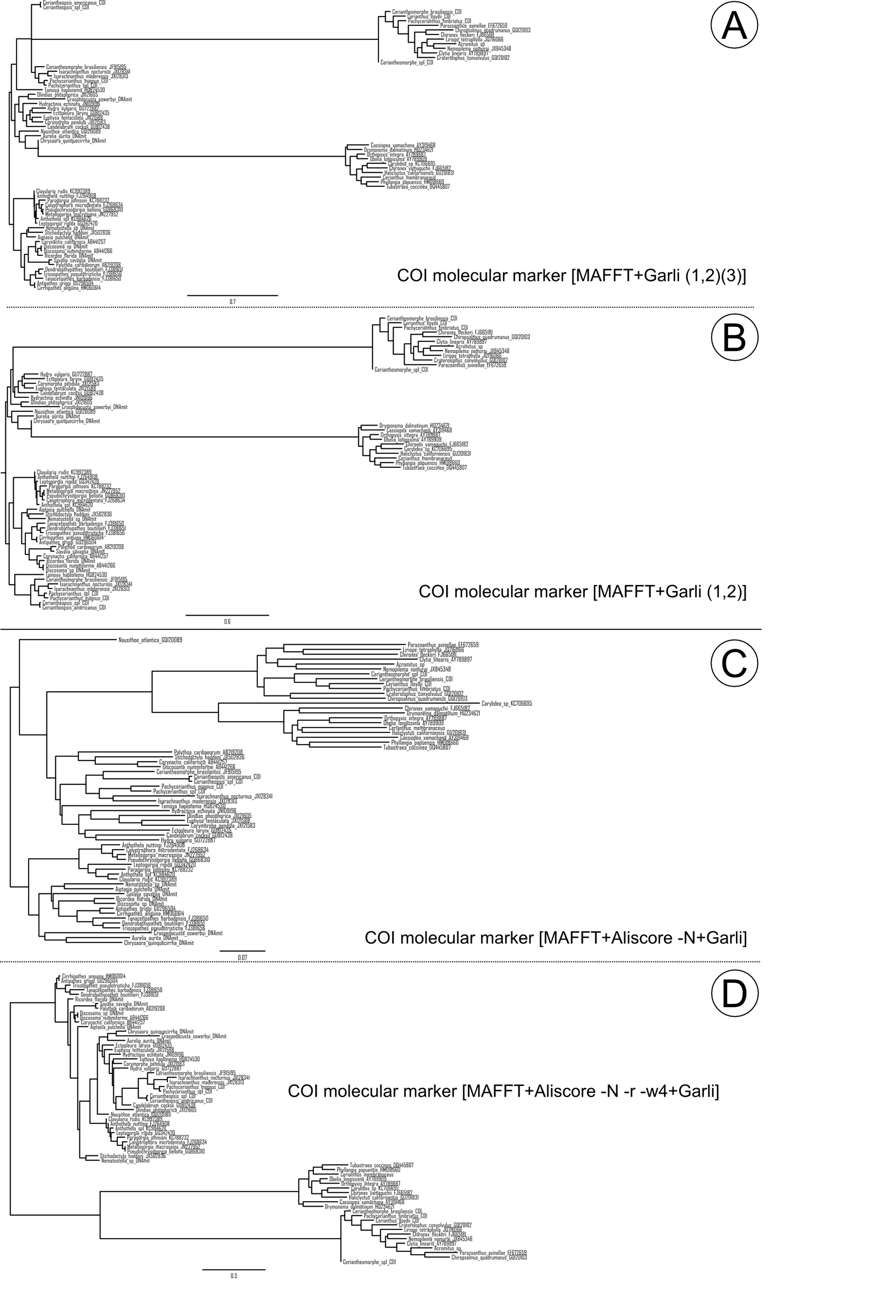

Supplement: Figure S3 — ML phylograms from different analysis datasets for cytochrome oxidase I (COI) cnidarian sequences estimated in Garli (100 replicates; [72] ). Partition analysis with PartitionFinder [73] defined two basic partitions as optimal to estimate gene phylogeny for COI (all codon positions): first and second position (partition 1, model TVM+G) and third position (partition 2, model SYM+G); then both partitions (Figure A) and partition 1 only (Figure B) were analyzed. Trying to overcome molecular saturation (non-phylogenetic related heterogeneity), the COI dataset was filtered at two different intensity levels: treating gaps as missing data (“Aliscore -N” strategy; Figure C) and a more intense approach (“Aliscore -N -r -w4”; Figure D). The COI dataset (Genbank IDs presented in terminal’s names) was originally aligned in MAFFT (codon frame checked); the root position was defined a posteriori (random position; no effect on ML analysis). (TIF) [file pone.0086612.s003.tif]
