# Supplementary material for: Fast-Evolving Mitochondrial DNA in Ceriantharia: A Reflection of Hexacorallia Paraphyly?
Source: PLoS One. 2014 Jan 27;9(1):e86612. doi: 10.1371/journal.pone.0086612 (PMC3903554; doi:10.1371/journal.pone.0086612)
Supplement: Text S1 — Discussion on evolution and previous systematic considerations of Ceriantharia and Anthozoa. (DOCX) [file pone.0086612.s007.docx]

**SUPPLEMENTARY MATERIAL**

**SM 1**

**Phylogenetic position of Ceriantharia**

Molecular analyses that include sequences of Ceriantharia began in 1995, with Chen et al. (1995). In this paper the authors describe a "strange" result regarding the sequence of 28S ribosomal DNA, *Cerianthus* sp. (*Pachycerianthus magnus*). In this analysis the branch that represents Ceriantharia fell between the outgroup (cubozoan *Chironex fleckeri*) and the remaining Anthozoa. The authors argued that the position of Ceriantharia was not well understood and did not to delve into details. Still, the analysis demonstrated the inexistence of the proposed group Ceriantipatharia. Subsequently, Song & Won (1997) presented an analysis based on 18S ribosomal DNA sequences. The authors showed Ceriantharia as an independent clade sister of the clade Octocorallia+Hexacorallia. Berntson et al. (1999) conducted an analysis with two species of Ceriantharia; this is the first publication that utilized more than one sequence of Ceriantharia in the same analysis. However, the sequences used in this article were very different from any of those we obtained here, even for the same species, and comparison between this earlier study and the present one is impossible. Still, the authors note that the position of Ceriantharia was not consistent and this clade appeared in different positions depending on the tree. Two subsequent studies, Won et al. (2001) and Daly et al. (2003), aimed at an integrated morphological and molecular analysis of Anthozoa. However, the results regarding Ceriantharia still remained inconsistent. The most recent analyses that included Ceriantharia were conducted by Collins et al. (2006). In that article the maximum likelihood analyses showed Ceriantharia as another, independent subclass of Anthozoa.

The data presented in the current study indicate congruence with the results of Chen et al. (1995) and Won & Song (1997). On the basis of sequences obtained in this study the group Ceriantharia+Hexacorallia exists only with the addition of Octocorallia. However, bootstrap support for these results was low. Finally, a recent cnidarian phylogenetic analysis was presented based on several mitochondrial markers (protein coding genes, including COI). As part of its main results, (i) Anthozoa was paraphyletic, with Octocorallia as sister group of Medusozoa (a similar result in others mitochondrial based studies: Park et al. 2012, and partial results in Shao et al. 2006; Kayal et al. 2008; Lavrov et al. 2008); and (ii) the only cerianthid species sampled (*Ceriantheopsis americanus*) were placed as sister to the rest of Hexacorallia. However, the position of Ceriantharia is considered as unstable and not well defined by the authors (“Our data do not resolve the position of Ceriantharia (tube anemones”). Besides the different type of analysed data in these studies (mitochondrial vs nuclear genes), different outgroups were also utilized. This is not a trivial consideration, as the Ceriantharia lineage is a basal one in all main results, and it is a well recognized issue that outgroup choice can affect basic relationships in basal lineages.

**References**

Berntson EA, France SC, Mullineaux LS. 1999. Phylogenetic relationships within the class Anthozoa (phylum Cnidaria) based on nuclear 18S rDNA sequences. Mol Phylogenet Evol. 13:417-433.

Chen CA, Odorico DM, Lohuis M, Veron JEN, Miller DJ. 1995. Systematic relationships within the Anthozoa (Cnidaria: Anthozoa) using the 5´-end of the 28S rDNA. Mol Phylogenet Evol. 4:175-183.

Collins AG, Schuchert P, Marques AC, Jankowski T, Medina M, Schierwater B. 2006. Medusozoan phylogeny and character evolution clarified by new large and small subunit rDNA data and an assessment of the utility of phylogenetic mixture models. Syst Biol. 55:97-115.

Daly M, Fautin, D.G., Cappola VA. 2003. Systematics of the Hexacorallia (Cnidaria: Anthozoa). Zool J Linn Soc. 139:419-437.

Kayal E, Lavrov DV. 2008. The mitochondrial genome of *Hydra oligactis* (Cnidaria, Hydrozoa) sheds new light on animal mtDNA evolution and cnidarian phylogeny. Gene 410:177–186.

Lavrov DV, Wang X, Kelly M. 2008. Reconstructing ordinal relationships in the Demospongiae using mitochondrial genomic data. Mol Phylogenet Evol. 49:111– 124.

Park E, Hwang D-S, Lee J-S, Song J-I, Seo T-K, Won Y-J. 2012. Estimation of divergence times in cnidarian evolution based on mitochondrial protein-coding genes and the fossil record. Mol Phylogenet Evol. 62:329–345.

Shao Z, Graf S, Chaga OY, Lavrov DV. 2006. Mitochondrial genome of the moon jelly *Aurelia aurita* (Cnidaria, Scyphozoa): A linear DNA molecule encoding a putative DNA-dependent DNA polymerase. Gene 381: 90-101.

Song J-I, Won JH. 1997. Systematics relationship of the anthozoan orders based on the partial nuclear 18S rDNA sequences. Korean J Biol Sci. 1:43-52.

Won JH, Rho BJ, Song JI. 2001. A phylogenetic study of the Anthozoa (phylum Cnidaria) based on morphological and molecular characters. Coral Reefs 20:39-50.
